# Second harmonic generation hotspot on a centrosymmetric smooth silver surface

**DOI:** 10.1038/s41377-018-0053-6

**Published:** 2018-08-15

**Authors:** Matan Galanty, Omer Shavit, Adam Weissman, Hannah Aharon, David Gachet, Elad Segal, Adi Salomon

**Affiliations:** 1 Department of Chemistry, BINA Nano Center for Advanced Materials, Bar-Ilan University, Ramat-Gan, Israel; 2 Attolight AG, EPFL Innovation Park, Building D, 1015 Lausanne, Switzerland

## Abstract

Second harmonic generation (SHG) is forbidden for materials with inversion symmetry, such as bulk metals. Symmetry can be broken by morphological or dielectric discontinuities, yet SHG from a smooth continuous metallic surface is negligible. Using non-linear microscopy, we experimentally demonstrate enhanced SHG within an area of smooth silver film surrounded by nanocavities. Nanocavity-assisted SHG is locally enhanced by more than one order of magnitude compared to a neighboring silver surface area. Linear optical measurements and cathodoluminescence (CL) imaging substantiate these observations. We suggest that plasmonic modes launched from the edges of the nanocavities propagate onto the smooth silver film and annihilate, locally generating SHG. In addition, we show that these hotspots can be dynamically controlled in intensity and location by altering the polarization of the incoming field. Our results show that switchable nonlinear hotspots can be generated on smooth metallic films, with important applications in photocatalysis, single-molecule spectroscopy and non-linear surface imaging.

## Optics: Combining photons leads to new sensing and imaging technologies

Scientists have used nano-sized cavities etched onto a sliver surface for combining two photons to produce light with twice the frequency, opening the door for new applications in photo-catalysis, and sensing and imaging technologies. Second harmonic generation (SHG) is a process in which two photons with the same frequency combine to generate a photon with double the frequency. However, SHG cannot be produced in smooth continuous metallic surfaces unless the symmetry can be broken by abrupt changes in the material’s geometry or through the application of an electromagnetic field. Now, Adi Salomon and colleagues from Bar-Ilan University in Israel, in collaboration with Swiss researchers, have produced SHG from the flat area between pairs of triangular nanocavities etched onto a smooth silver surface, and could see use in photo-catalysis, non-linear surface imaging and single-molecule spectroscopy.

## Introduction

Second harmonic generation (SHG) is a process in which two photons at the fundamental frequency combine to generate a photon with a double frequency through nonlinear scattering^1^. SHG is forbidden in materials with inversion symmetry such as bulk metals, yet symmetry can be broken by material discontinuity^2,3^ or by electromagnetic (EM) field properties^4,5^. Significant SHG responses were observed from metallic surfaces and were attributed to nonlinear polarizability of free and bound electrons at the metal interface^6^.

Plasmonic structures, whether particles or cavities, increase the nonlinear response dramatically due to their enhancement of the EM fields at the interface^7–15^. Nonlinear responses of both nanoparticles and nanocavities with various sizes and shapes, including metasurfaces, were studied both in the visible and near-infrared regimes^8,9,14,16–22^. Additionally, metallic nanoparticles have been shown to increase the SHG responses of dielectric particles^23,24^. Typically, SHG emanating from nanostructures is enhanced when the plasmonic structure resonates with the electric field, *E*, either at the fundamental (ω) or harmonic frequency (2ω) or both^25–29^, since the SHG intensity is proportional to (|*E*(*ω*)|^2^|*E*(2*ω*)|)^2^ ^30–32^. While most studies have focused on metallic structures with no inversion symmetry, SHG is also observed from symmetric plasmonic structures^21,33^. The reason for such an enhanced nonlinear conversion process has remained elusive.

Nanocavities milled in a metallic film have emerged as a promising class of optical materials^19,20,34–36^. The localized modes of the nanocavities may lead to the excitation of surface plasmon polaritons (SPPs), and long-range coupling between cavities can occur^13,17,19,20,37,38^. In this respect, nanocavities differ from their so-called “complementary” nanoparticle structures and they exhibit dissimilar optical behavior. Elementary SPPs excited from nanocavities can combine and emit a more energetic photon^39^. In this case, SPPs do not boost local fields but are rather annihilated to form a photon at double the frequency. This implies that in practice, a sub-wavelength aperture is not necessarily needed to generate enhanced SHG. This was experimentally shown by Woggon and co-workers when measuring SHG emission from silver films using a Kretschmann configuration^40^ and theoretically supported by Finazzi and Ciccacci^39^.

**Fig. 1:**
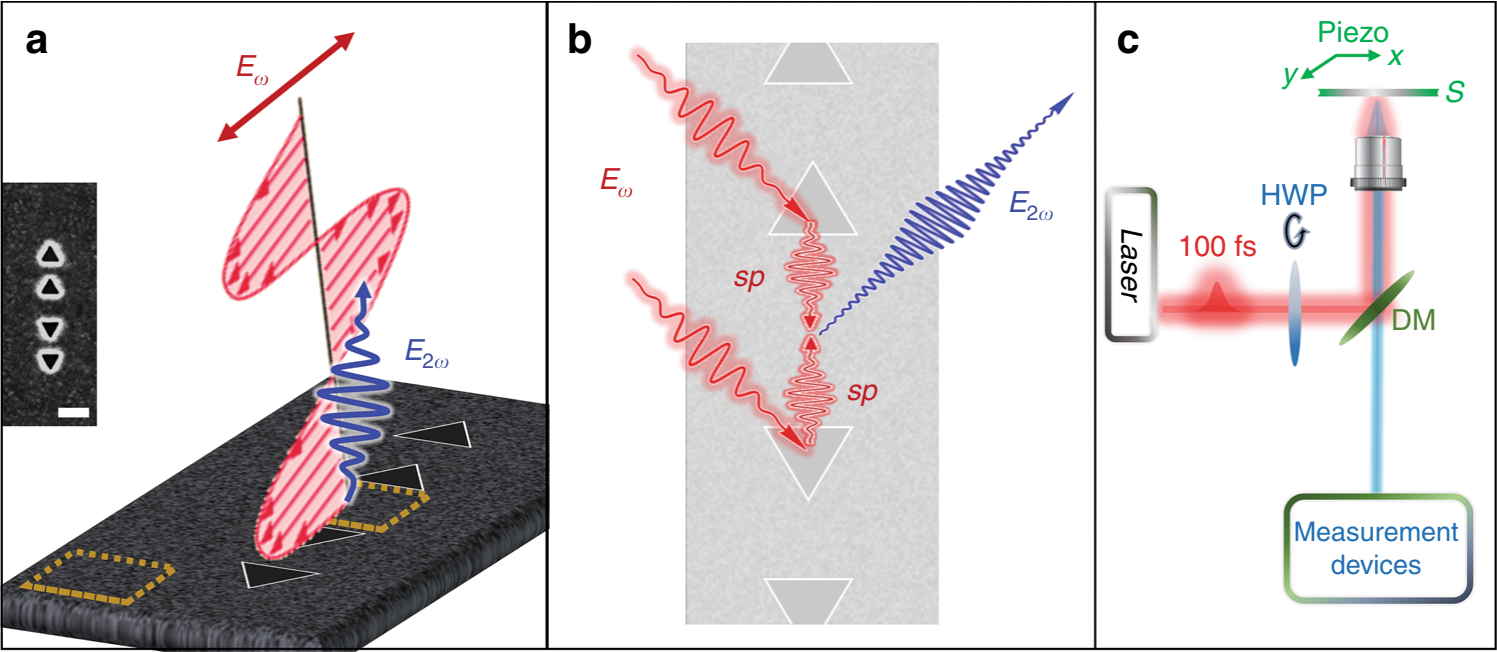
Schematic representation of the studied plasmonic structure and the second-harmonic mechanism emanating from the flat surface area between the cavities. **a** The plasmonic structure is composed of two sub-units, i.e., two pairs of triangular nanocavities with a side length of 200 nm milled into a silver film. Their base-to-base separation is 400 nm (inset left middle). The whole plasmonic structure is illuminated with linearly polarized light at a normal incident angle, with a spot size of approximately two microns. The enhanced second harmonic signal emanates from the flat area confined between the two sub-units (indicated by a dashed square), while the response from the neighboring surface is negligible (square at the bottom left corner). **b** A broadband plasmonic mode is excited by the fundamental field at the triangular cavity side length. Those plasmonic modes propagate onto the surface towards each other and annihilate to form a photon at twice the frequency, which is detected in the far field, without the need for sub-wavelength apertures to scatter the field. The studied plasmonic structure exhibits inversion symmetry. **c** The sample is excited in a reflection mode through an objective, and the SHG is collected through the same objective. Both the plasmonic structures and the silver surface are scanned at the same measurement, enabling the comparison of the emanating signal and verification of the alignment of the set-up. For a full description, see S2.2 and Fig. S2

Here, we experimentally demonstrate enhanced SHG emission from a smooth silver film area surrounded by several nanocavities. We used triangular nanocavities milled into a silver thin film in a configuration with a base-to-base distance of 400 nm. We show that SHG emission results solely from the strong interaction between the SPP modes propagating from each cavity edge. Those SPPs, at the fundamental frequency, propagate and annihilate to form a spatially confined SHG “hotspot” on the flat surface (Fig. 1). In this geometry, SHG stemming from the inter-cavity smooth surface is dramatically enhanced, and that from the triangular plasmonic cavities is suppressed. These observations and their interpretation are further supported by spatially resolved linear transmission measurements, as well as by CL micro-spectrometry.

## Materials and methods

Our sample is composed of two pairs of triangular cavities. The triangular nanocavities have a typical side length of 200 nm, and they are fabricated using a focused ion beam (FIB, Dual Beam, Helios Nano Lab 600i from FEI), in a 200-nm-thick smooth silver film deposited on a clean fused silica substrate (Fig. 1 and Fig. S1). The silver surface is covered by a 150-nm-thick polyvinyl alcohol (PVA) layer in order to match the refractive indices on both sides of the sample (average refractive index in the visible to near-infrared region on the order of 1.5) and to prevent sample oxidation and aging.

Similar triangular cavities have been thoroughly studied and were found to enhance nonlinear responses such as SHG. Their geometry with respect to the fundamental incident field plays an essential role in governing the optical response^12,13,41^. A triangular cavity supports several plasmonic modes, among them a broadband side-length mode, which can launch SPP modes onto the surface^19,20,42,43^. Based on these earlier findings, we designed a plasmonic structure in which the “bases” of two adjacent triangular cavities are face-to-face, a configuration that leads to a confinement of the EM field onto the flat surface between the cavities and the frequency of which can be adjusted by the distance between the cavities^16^. The SHG response from such structures was investigated using custom-fabricated two-photon microscopes (Fig. 1c and Fig. S2). The samples were excited in reflection mode at normal incidence by a tunable (700–1000 nm) Ti:Sapphire laser (Spectra-Physics Mai-Tai HP, 100 fs, 80 MHz), using a ×50 numerical aperture (NA) 0.5 air objective lens (Olympus), giving an illumination spot size of approximately 2 microns. SHG was collected through the same objective lens. The SHG images were scanned at 940 nm using a piezo stage (Piezosystem, Jena). The piezo stage spatial resolution is of a few nanometers, and our system spatial resolution is approximately 250 nm, a sub-diffraction limit resolution. The polarization-dependent measurements were performed by varying the polarization angle of the fundamental field using a rotating half-wavelength plate. Prior to the measurements, the system was carefully aligned, and the response from the silver surface was found to be negligible. In addition, each time, we ran rotational SHG measurements (polar plots) from several regions on the silver surface to verify that the fundamental field was at normal incidence and that the system was well aligned. Our results have been repeated more than ten times on different structures and samples.

## Results and discussion

**Fig. 2:**
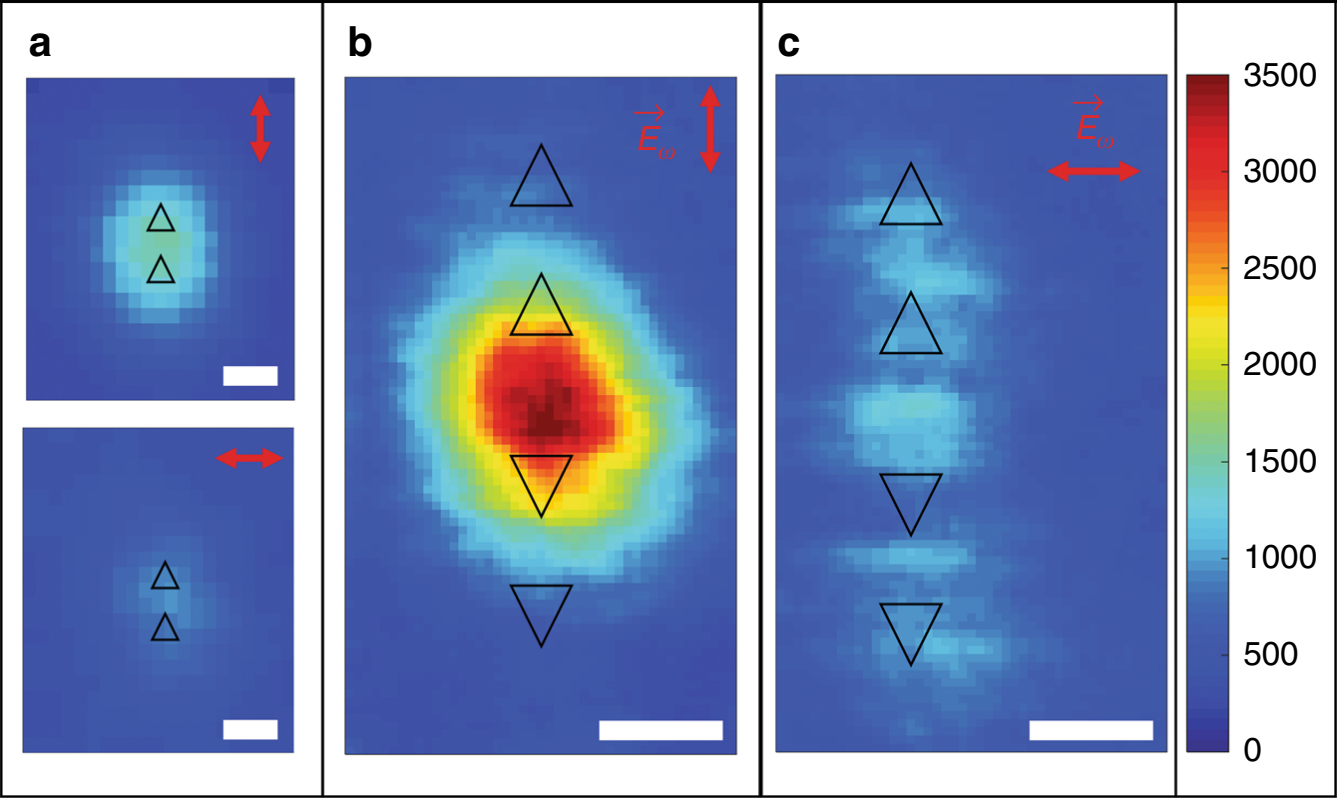
SHG from a flat centrosymmetric silver film. **a** SHG imaging of the basic sub-unit (Fig. 1a), a pair of two triangular cavities, under two orthogonal polarizations. When the polarization of the incoming field is along the interaction axis (in-axis), an enhanced SHG signal is observed in contrast to the orthogonal polarization (top and bottom left, respectively). **b**, **c** SHG response from our plasmonic device, composed of two sub-units facing each other. **b** When the polarization state of the incoming field is along the interaction axis, the SHG emanates from the silver surface area between the cavities rather than from the sub-units as in panel **a**. **c** When the polarization of the incoming field is orthogonal to the interaction axis, the SHG response is weak and is distributed along the entire plasmonic structure. The scale bar is 400 nm for each of the images. The triangular cavities are drawn to size to guide the eye. The wavelength of the fundamental field in all measurements is 940 nm, and the color bar is in counts per second (CPS) and is valid for all the presented figures

When the two triangular cavities are arranged in a base-to-tip configuration, an intense SHG signal emanates from them (Fig. 2a). The SHG response is half an order of magnitude stronger for the in-axis (interaction axis) polarization compared to the off-axis (orthogonal) polarization of the incoming field, in agreement with previous work^13^. Both cavities emit SHG in phase and are considered to be strongly coupled. They are referred to here as a sub-unit. Now, when two of these sub-units are arranged base-to-base, the overall optical response is dramatically changed as shown in (Fig. 2b, c). When the polarization of the incoming field is along the symmetry axis, the SHG is enhanced and is emitted solely from the area in between the two sub-units. In turn, the SHG response of the outer cavity pairs drops dramatically compared to the earlier case and is comparable to that of a free silver surface (see also Fig. S3). As before, when the polarization state of the incoming field is orthogonal to the symmetry axis, the SHG response of the plasmonic unit is weak and spreads uniformly across the entire unit. Clearly, the two sub-units together with the silver surface behave as a single entity, and the area from which SHG is detected is smaller than that the sum of the two sub-units (Fig. 2). The SHG emission and its dependency on the fundamental wavelength are shown in the supporting information (Fig. S3b). We note as well that the surface between the cavities is ultra-smooth and there is no sub-wavelength aperture to scatter the plasmonic modes to the free-propagating light (see S1). In fact, when the surface is rough, we do not observe this phenomenon. The SHG nature of the structure under study is confirmed by spectral analysis (Fig. S4). Finally, we measured the two sub-units in a bowtie configuration, *ceteris paribus*. As expected, the two sub-units behave as individual units with no coupling between them (Fig. S5), and no SHG is detected from the flat surface between them.

**Fig. 3:**
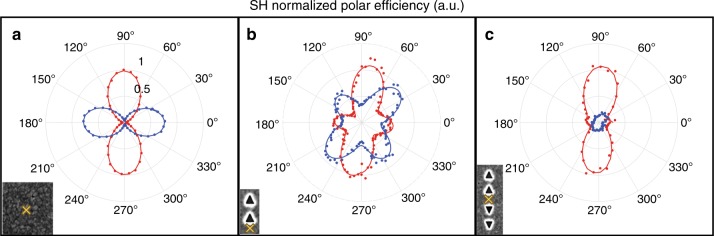
Polarization-dependent SHG response of the plasmonic system. The *x*-axis (blue) and *y*-axis polarization (red) components of the SHG signal are collected in reflection mode as a function of the angle of the incoming field polarization: **a** from a silver film, showing an incoherent second-harmonic response (HRS); **b** the same, from a Ag substrate with a sub-unit, displaying an octupolar signature with a moderate dipolar signature along the *y*-axis (red plot); and **c** from the flat surface between the two sub-units, showing a very strong dipolar behavior. The harmonic responses at 2*ω* are normalized to the response of the silver film (see S7). It can be seen that the non-linear response in **c** is enhanced by an order of magnitude compared to a silver thin film. The yellow markers in the inset images represent the center position of the driving fundamental field. The studied samples were excited at a power of 2.5 mW at 940 nm, and the bare surface was excited with 25 mW

How large is the SHG enhancement observed between the base-to-base configurations compared to a smooth bare-silver surface? The effective nonlinear coefficient for such an arrangement is 8.9 × 10^−15^ W^−1^ (for detailed calculations, see S6 and table S1), seven-fold greater than the hyper-Rayleigh scattering (HRS) from a neighboring bare-silver film. Thus, for a laser power of 4 mW and a NA 0.5 objective, we obtain 3.5 × 10^4^ photons at 2ω (for detailed calculations see S6 and table S1). We note that this high gain is achieved on a flat surface without a demanding fabrication. It is now clear that the nano-patterned silver surface plays an important role in achieving such a dynamic SH hotspot. To better understand the nature of the interaction between the sub-units and the surface, we run a full polarization analysis of the SHG emission generated by the structures composing the plasmonic unit. The two orthogonal polarization components of the SHG output emission are individually measured as a function of the polarization angle of the fundamental field. In this way, we directly obtain information about the second-order susceptibility tensor *χ*^(2)^_*ijk*_. Figure 3 shows the polar plots corresponding to each sub-unit composing the full studied structure. As expected, in the case of the bare-silver film, we observed two perpendicular dipolar patterns (Fig. 3a). They are due to HRS, the incoherent summation of contributions from randomly oriented dipoles, associated with symmetry-breaking silver grains and grain boundaries^12,30^. Next, for the pair of two equally aligned oriented triangular cavities, the off-axis shows an octupolar polarization behavior, the signature of a three-fold symmetric object^11,12,43–45^, while the in-axis reveals moderate coupling between the two triangular cavities. A highly polarized SHG emission is observed for the full structure composed of two oppositely oriented sub-units (Fig. 3c). Particularly, only the *y*-axis output is appreciable, whereas the *x*-axis is close to the noise level. The *y*-axis component displays a residual octupolar symmetry, indicating that the tensor element of the triangular cavity building blocks does not completely vanish. The polar plots shown in Fig. 3 are all normalized with respect to the SHG response of a flat silver film (see S7). It can be seen that the emission stemming from the flat area of the plasmonic structure is about one order of magnitude higher than that of a bare-silver surface, in agreement with the data shown in Fig. 2.

**Fig. 4:**
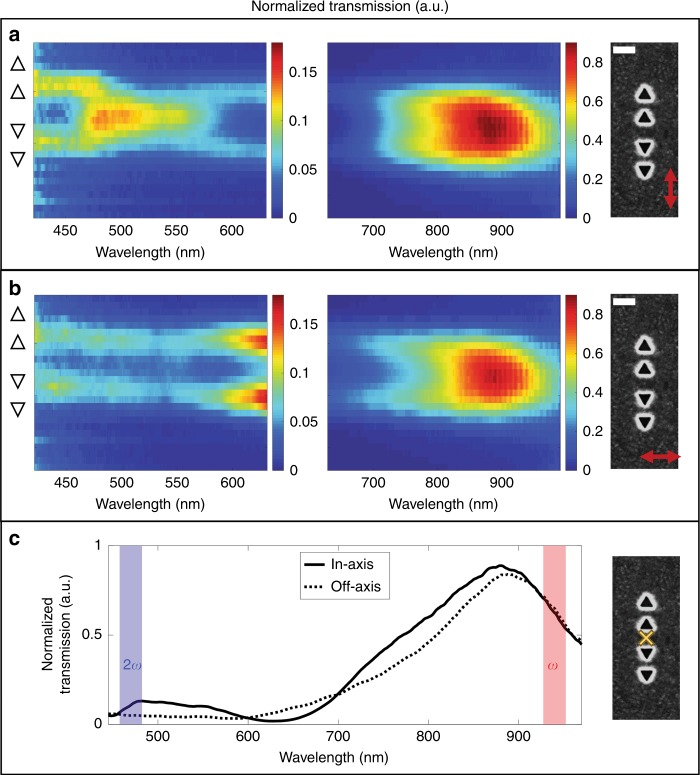
Linear transmission spectra of the full plasmonic device. **a** Spatially resolved linear transmission spectra of the plasmonic structure for a driving field polarized parallel to the interaction axis of the plasmonic structure. The plasmonic structure is shown in the right part of the inset images, and the spectra shown are a function of the position (*y*-axis) of the plasmonic structure. A dominant transmission peak at approximately 880 nm is observed together with an additional peak at ~500 nm (left image). It is worth noting that the peak at 500 nm emanates from the middle of the plasmonic structure rather from the sub-units. When the polarization is orthogonal to the interaction axis (**b**), the transmission peak at approximately 500 nm vanishes, and a weak broadband peak emanates from each of the sub-units. On the left side of the transmission images, the triangular cavities are illustrated. The SEM image on the right side is to scale with the transmission map for **a**–**c**. Spectra are taken from the center of the spatially resolved transmission spectra for two orthogonal polarizations. Red and blue stripes indicate the fundamental and second harmonic wavelengths. The yellow marker on the SEM image indicates the area from which the spectra were taken

To further characterize the behavior of this plasmonic structure, we studied the linear spectral transmission of the system, using a polarized broadband light source (see supporting information S2.2 for the setup information). The results are shown in Fig. 4. For both polarizations, an 885 nm peak that is delocalized over the entire structure appears, while a weaker and broader peak at ~450–480 nm is observed only around each sub-unit. However, for the polarization parallel to the interaction axis (Fig. 4a), an additional, high-intensity peak, centered at ~500 nm, is observed. This peak is localized between the two sub-units, on the bare-silver region. This mode arises from the coupling between the triangular side length mode and the bare-silver surface, as shown recently^19^. The appearance of this mode gives rise to a high density of photonic states in this spectral range in accordance with the observed enhanced SHG emission. When the polarization of the incoming field is oriented perpendicularly to the interaction axis, this peak vanishes completely, and transmission occurs only from the cavities themselves rather than from the bare-silver area between the two sub-units (Fig. 4b). The linear transmission spectra taken at the level of the hotspot area for two orthogonal polarizations are shown in Fig. 4c. A double resonance behavior is observed for in-axis polarization, unlike the off-axis case, in agreement with the observed SHG results.

**Fig. 5:**
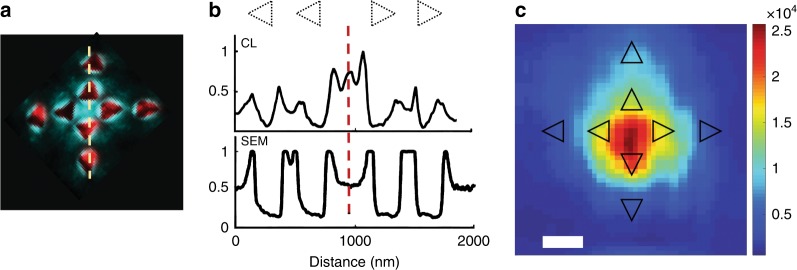
Cathodoluminescence map and SHG scan of the quadrupolar structure. **a** CL map at two wavelengths, 650 ± 25 nm (red), and 370 ± 25 nm (blue). The 650 nm emission emanates from the cavities, while the 370 nm emission emanates from the center of the structure between the cavities. A blue color spot is observed at the center of the structure (see also Fig.S6). An intensity profile at a wavelength of 370 nm, along the yellow dashed line, is shown in **b** alongside the SEM cross section (down) of the structure. A clear, intense and narrow mode is observed at ~370 nm from the flat area in the middle of the structure (see the red dashed line). Four triangles are added to guide the eye. **c** The SHG from the quadrupolar structure emanates from the flat surface area in the middle of the structure. Triangles (to scale) are added to guide the eye. The scale bar length is 400 nm, and the color bar is in counts per second (CPS)

To further support this claim and to map these modes with increased spatial resolution, we also performed cathodoluminescence (CL) measurements on a similar plasmonic structure with identical geometrical parameters. In CL, the plasmonic modes are locally excited by an electron beam, and spatial mapping of the emitted photons at a given wavelengths directly maps the plasmonic modes of the system^46–48^ (see S 2.3 for experimental details). Hence, the radiative plasmonic response of the metallic system can be probed, and high-resolution spectral imaging can be achieved^49–51^. In contrast, the  excite mechanism in CL is different from that of SHG in that both probe the plasmonic response and the optical properties of the plasmonic modes. Figure 5a shows a pseudo-colored CL image of the plasmonic structure from which the PVA layer has been removed (metal–air interface). The colors correspond to the fundamental (red) and SHG (blue) modes. The cavities show a strong response at the fundamental frequency, while a robust response at 2ω is observed in their close vicinity and in the middle of the structure^19,20^. The wavelength of interest in the CL measurements is 370 nm, correlated with the 470 nm in the SHG measurements due to differences in the refractive indices, i.e., PVA vs. air. Figure 5b shows the CL cross section along the structure at 370 nm alongside the SEM cross section. Beside the detected cavity modes, a confined and spectrally narrow mode is observed in the central flat area at ~370 nm (see also Fig.S6) in agreement with the transmission measurements shown above. The SHG emission from this plasmonic structure is shown in Fig. 5c. Clearly, the SHG is emanating from the smooth surface between the plasmonic cavities in accordance with the above observation. We note that such a structure is highly symmetric and that the silver surface between the cavities is smooth, with no sub-wavelength apertures. Otherwise, no CL or SHG are observed from the center (see supporting information Fig. S7).

## Conclusions

In this study, we demonstrate an enhanced SHG response from a centrosymmetric nanostructured silver film. We observed a very strong SHG signal from the smooth surface area between the triangular cavities, where the amplitude of the SHG signal is highly dependent on the polarization state of the driving fundamental field relative to the orientation of the triangular cavities. Based on several observations, we suggest that this strong SHG response arises from SPP modes launched from adjacent sub-units and subsequent plasmon–plasmon annihilation on the flat surface area: (i) in a base-to-base configuration and for in-axis polarization of the incoming field, the SHG emission emanates from the flat surface between the cavities, whereas the signal from the subunits is dramatically reduced. Individually, those sub-units enhance SHG (Fig. 2). This observation would not be expected for a classical SHG process; (ii) a result conforming to this classical expectation is observed for the same number of subunits, but in a bow-tie configuration (Fig. S5) or when the surface is rough (Fig. S7). This indicates that the phenomenon is due to the propagation of surface plasmons rather than localized plasmonic modes of the cavities; (iii) throughout this study, we detect SHG in the far field using an objective with an NA of 0.5. Thus, we would not expect to observe SHG between the cavities unless sub-wavelength apertures were present to couple the nearfield SHG to the far-field. Even so, we observed a localized enhanced SHG signal in the absence of such sub-wavelength apertures because plasmon–plasmon annihilation creates photons propagating within our detection NA; and (iv) we note that although the main studied structure is elongated, the SHG emission is rather symmetrical, spatially spreading equally along the *x*- and the *y*-axes.

In previously studied systems^8,12,13,36,41,52–54^, nanocavities were used to boost the EM fields resulting in an enhanced nonlinear response. In stark contrast, the symmetric plasmonic systems introduced herein, with inversion symmetry, enhance SHG emission through what we believe to be a plasmon–plasmon annihilation processes.

One might consider the assembly of the cavities in the framework of the extended hybridization model. That is, the cavities are in analogous to the atoms and the SHG emanating we observed from the middle could be the probability function. However, the mode is too localized to support this explanation.

From an application point of view, our findings may further be used to produce SHG hotspots at any given location on a given metallic surface. The ability to form such a strongly confined SHG spot in the optical regime may provide a useful tool to study physical-chemical transformations occurring on surfaces, such as photocatalysis processes. Moreover, it may be useful for molecular, label-free biological sensing and imaging near surfaces^55^.

## Electronic supplementary material

Second Harmonic Generation Hot-Spot on a Centrosymmetric Smooth Silver Surface
