# Supplementary material for: Second harmonic generation hotspot on a centrosymmetric smooth silver surface
Source: Light Sci Appl. 2018 Aug 15;7:49. doi: 10.1038/s41377-018-0053-6 (PMC6107033; doi:10.1038/s41377-018-0053-6)
Supplement: Supplementary file 1 — Second Harmonic Generation Hot-Spot on a Centrosymmetric Smooth Silver Surface [file 41377_2018_53_MOESM1_ESM.docx]

Second Harmonic Generation Hot-Spot on a Centrosymmetric Smooth Silver Surface

Matan Galanty^1‡^, Omer Shavit^1‡^, Adam Weissman^1^, Hannah Aharon^1^, David Gachet^2^, Elad Segal^1^, and Adi Salomon^1*^.

^‡^Equal contributions.

^*^Corresponding author: E-mail: adi.salomon@biu.ac.il, TEL: +972-3-738-4235, FAX: 972-3-7384053

^1^Department of Chemistry, BINA Nano center for advanced materials, Bar-Ilan University, Ramat-Gan, Israel.

^2^Attolight AG, EPFL Innovation Park, Building D, 1015 Lausanne, Switzerland.

**1. Sample preparation:**

Silver films of about 200 nm thickness were evaporated onto a clean fused silica substrate under high vacuum conditions, with a roughness of less than 2 nm. In order to get such high quality film the glass slides were cleaned before using Helmanex 1:100 (using DI water) and were sonicated for about 20 min. Then a 99.999% nitrogen flow was used to carefully dry the glass slides. Samples with high roughness of 5-10 nm were not used. Triangular nanocavities with a typical side length of 200 nm were fabricated by focused ion beam (FIB, FEI, Helios Nano Lab 600i). Normally we used a current of about 30 pA with about 60 passes. To match the refractive indices on both sides of the sample and to prevent sample oxidation, the silver surface was covered by a 150 nm thick polyvinyl alcohol (PVA) layer with an average refractive index in the visible to near-infrared of the order of 1.5. An uncoated sample SEM image is presented in Figure S1.

To emphasize that the SH emission is not due to scattering from an object or a defect in the flat area in between the cavities, we show a zoom-in image of this area in Figure S1(b).


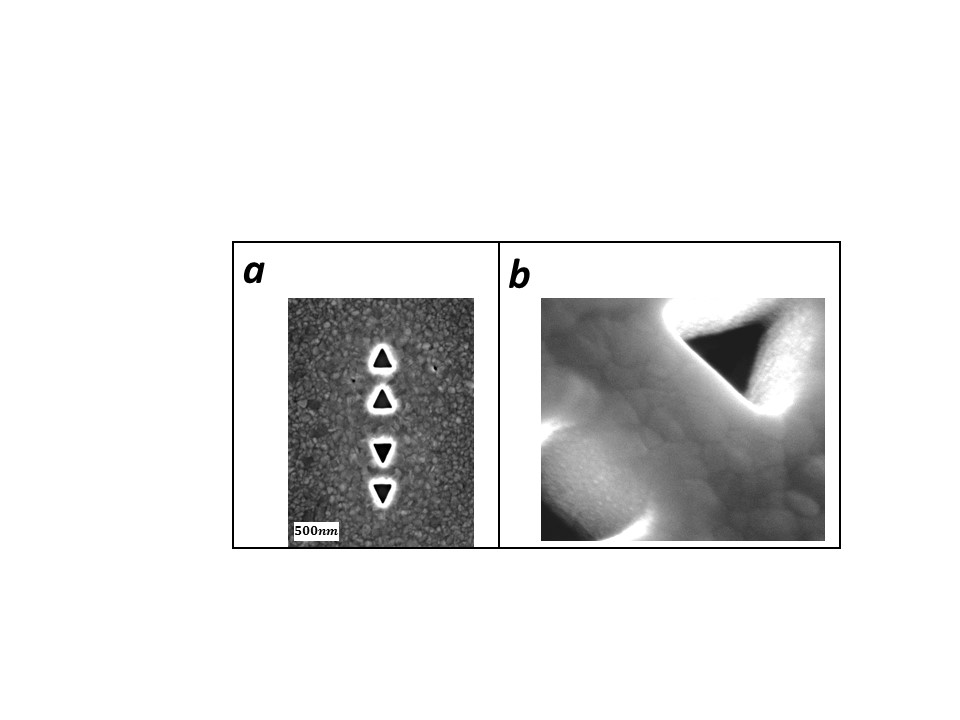


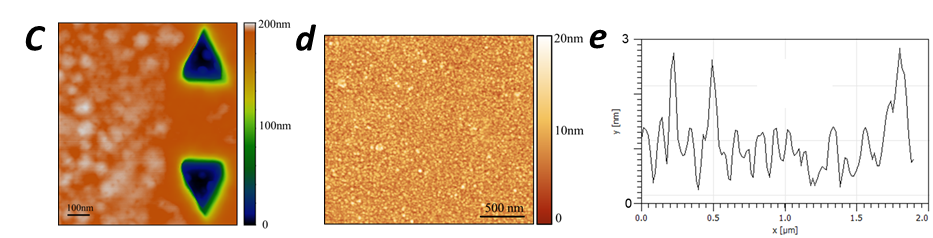


Figure S1: (a) SEM image of the sample showing two sub-units with pairs of triangular nanocavities in a base-to base configuration. The side length of triangles is 200 nm. The distance in between the two sub-units is 400 nm. (b) Close-up view of image (a). The scale bar is not shown, as the image is tilted. (c) atomic force microscopy (AFM) scanning of part of the structure showing that the surface between the triangular holes is smoother compared to other areas (d) AFM scanning of the silver film, showing that the film is relatively smooth, with its scan profile (right).

**2. Methods:**

2.1 Linear optical measurements:

Transmission spectra were recorded using an Olympus inverted microscope (IX83 series) coupled to a spectrophotometer (IsoPlane SCT-320, Princeton Instruments) and a charge-coupled device camera (CCD, PIXS1024b, Princeton Instruments). The samples were illuminated in a bright-field mode with a collimated polarized white-light source, and transmission spectra were collected with a 100x magnification (NA = 0.95) objective. The transmission spectra were normalized using the transmission spectrum of a glass substrate under the same optical conditions.

The transmission images have been taken by the same set-up, using the PIXS1024b, which contain 1024X1024 pixels. The images were taken in two orthogonal polarizations using the polarizer mounted in the microscope.

2.2 Second-harmonic (SH) measurements:

The samples were illuminated by a tunable Ti:Sapphire laser (Spectra-Physics Mai-Tai HP, 100 fs, 80 MHz, 690–1080 nm) at a **normal incident angle**. We mostly used a 940 nm illumination wavelength. The sample was scanned with a piezo stage (Piezosystem Jena) in a closed-loop feedback. The linearly polarized laser beam was focused on the sample using a 50x objective lens (NA = 0.5), and the SHG signal was collected by the same objective lens in reflection mode. The reflected light was filtered by a dichroic mirror (Chroma) and band-pass filters (Semrock), directed to a polarized beam splitter (PBS), and detected by two avalanche photodiodes (APD, PerkinElmer), or directed to a spectrograph (Shamrock 303i) equipped with an electron multiplier charge-coupled device (EMCCD) camera (Andor Newton) to collect the emitted spectrum. The polarization measurements were performed by varying the input polarization angle of the fundamental field using a rotating half-wavelength plate. The two branches of the PBS correspond to two orthogonal polarizations of the SHG signal emitted from the sample (along the *x* and *y* axes, *s-* and *p-*polarization).

The schematic illustration below shows the microscope-based setup.


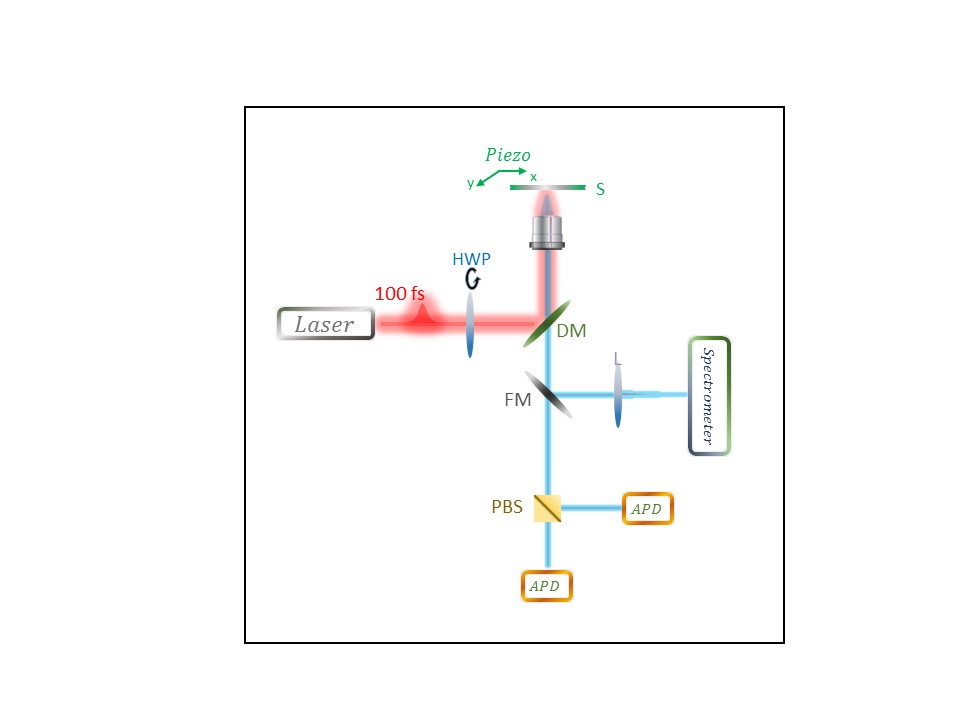


Figure S2: Schematic illustration of the SHG microscopy setup. The fundamental excitation polarization is rotated with a half-wave plate (HWP). A dichroic mirror (DM) is used to direct the light upward to the sample (S), which is mounted on a piezo actuator (Piezo) to allow high accuracy scanning. The DM maintain the phase of the polarization components, one can see that from the polar plot of HRS from silver surface (Figure 3a). The output SH beam is directed either to a spectrometer equipped with an electron multiplier charge-coupled device (EMCCD), or onto a polarizing beam splitter (PBS) coupled to two avalanche photodiodes (APDs), by using a flip mirror (FM)

2.3 Cathodoluminescence (CL) microspectrometry measurements:

CL measurements were performed on an Attolight Rosa 4634 CL microscope, which tightly integrates an achromatic reflective lens within the objective lens of a field-emission-gun scanning electron microscope (FEG-SEM). The focal plane of the light lens matches the FEG-SEM optimum working distance. The CL was spectrally resolved with a Czerny-Turner spectrometer and measured with a UV-vis CCD camera. The acceleration voltage and emission current of the electron beam were 7 kV and 20 nA, respectively. CL data were acquired in hyperspectral mode, namely a CL spectrum was acquired for each position of the electron beam on the sample. The integration time per spectrum was 100 ms. Note that unlike the linear transmission measurements, CL measurements are performed on a silver-air interface (uncoated samples), therefore the modes deduced from the CL measurements are blue-shifted (by a factor of ~1.5).

**3. SHG intensity cross section of the studied sample and wavelength dependency**

The intensity profile of the SHG signal emanating from the sample is shown in Figure S3, along with the triangular cavity positions. The SHG scanning was performed along 2.5 µm. The SHG emission stemming from the outer cavities (at *x* positions 0.5 and 2 µm) is comparable to that of a neighboring bare silver film (at *x* positions 0.2 and 2.3 µm for example). The SHG signal is emitted from an area with a radius of about 0.6 µm, using an NA of 0.5 and an illumination wavelength of 940 nm.

The wavelength dependency of studied structure (Figure 2) is shown in Figure S3(b). Several SHG scans have been taken on the same plasmonic structure, but with different fundamental wavelengths. Comparing the SHG scans of 880 nm and 940 nm, one can easily see that although the average signal is similar, the emission in the case of 940 nm is more localized and symmetric, unlike the elongated one for 880 nm. In fact, it seems that the resolution for 940 nm is better. We think that in the case of 880 nm, the localized mode in between the cavities is wiped out, and the majority of the SHG emission is steaming from the cavities themselves rather from than from the area between the cavities.


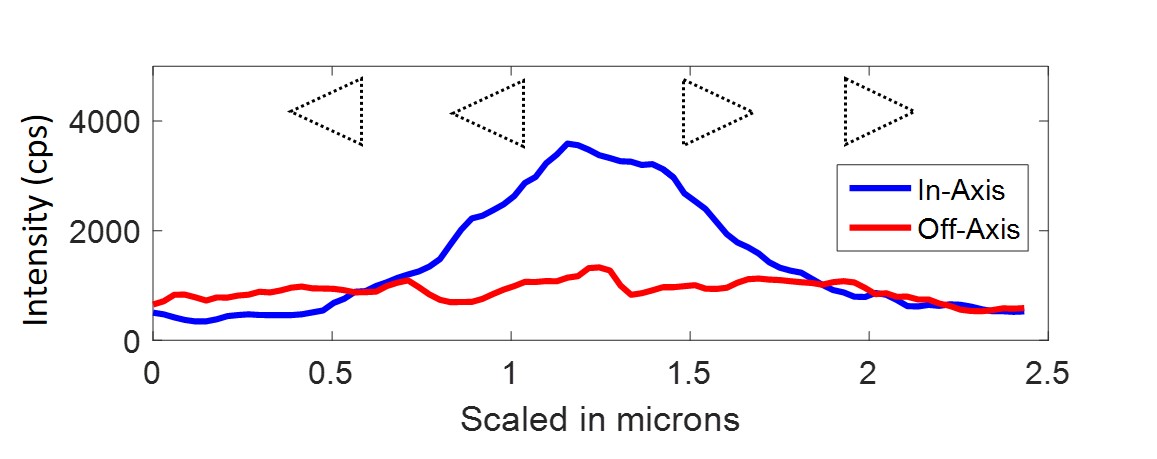
(a)

**
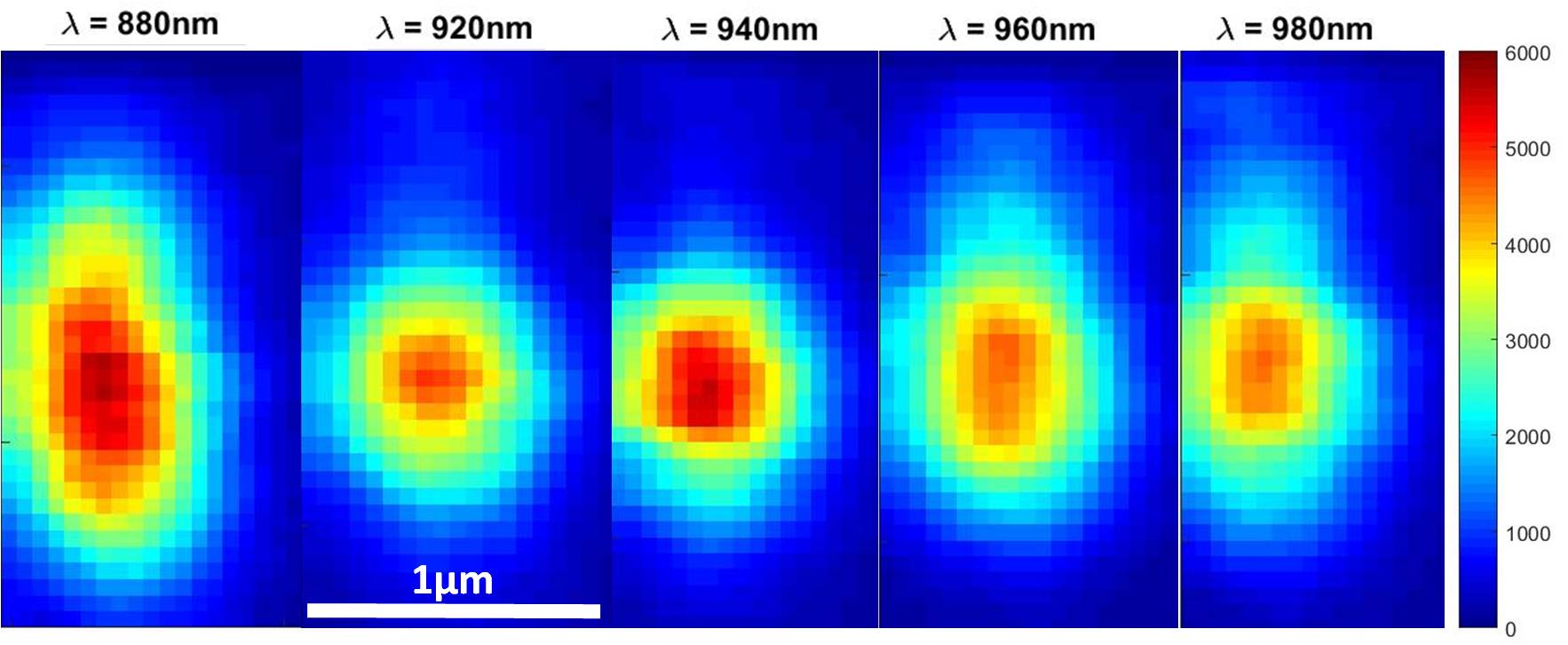
**(b)

Figure S3: (a) Cross sections of the SH scanning (Figures 2(b) and 2(c)) along the studied structure for two orthogonal polarizations. Sketches of the triangular cavities (in scale) are added to guide the eyes. When the polarization of the fundamental field is *in-axis* (blue), the SHG emanates from the middle of the structure onto the flat area in between the nanocavities. When the polarization is *off-axis* (red) the intensity is dramatically reduced and spreads along the entire structure. (b) SHG wavelength dependency of the studied structure (Figure S1a). All the parameters are the same but the fundamental wavelength. Apparently the spot size is smaller for 920 nm, 940 nm as compared to 880 nm for example, indicating that the resolution of the system does not necessary scales with the wavelength.

**4. Dependence of the SHG emission on input power:**

The SH emission should follow a quadratic behavior as a function of the incident power. A log-log plot is expected to show a linear dependence with a slope *m=*2 according to the following equation:

$$log\left( I_{2\omega} \right)=log\left[ \left( \varepsilon_{0}{\chi_{eff}}^{\left( 2 \right)} \right)^{2} \right]+2log\left( I_{\omega} \right)$$

Experimentally, the excitation and detection areas are the same as we work in reflection mode. Therefore, the same relationship applies for the power:

$log\left( P_{2\omega} \right)=log\left[ \left( \varepsilon_{0}{\chi_{eff}}^{\left( 2 \right)} \right)^{2} \right]+2log\left( P_{\omega} \right)$. Figure S4 shows experimental values of the SH power$\left( P_{2\omega} \right)$ as a function of the input power. We measured the SHG signal for the bare silver film (black line) and for the studied structure for two orthogonal polarizations of the fundamental beam, as mentioned in the legend. The interception point of these curves with the ordinate axis indicates the SHG efficiency, or the absolute value of the effective nonlinear term.


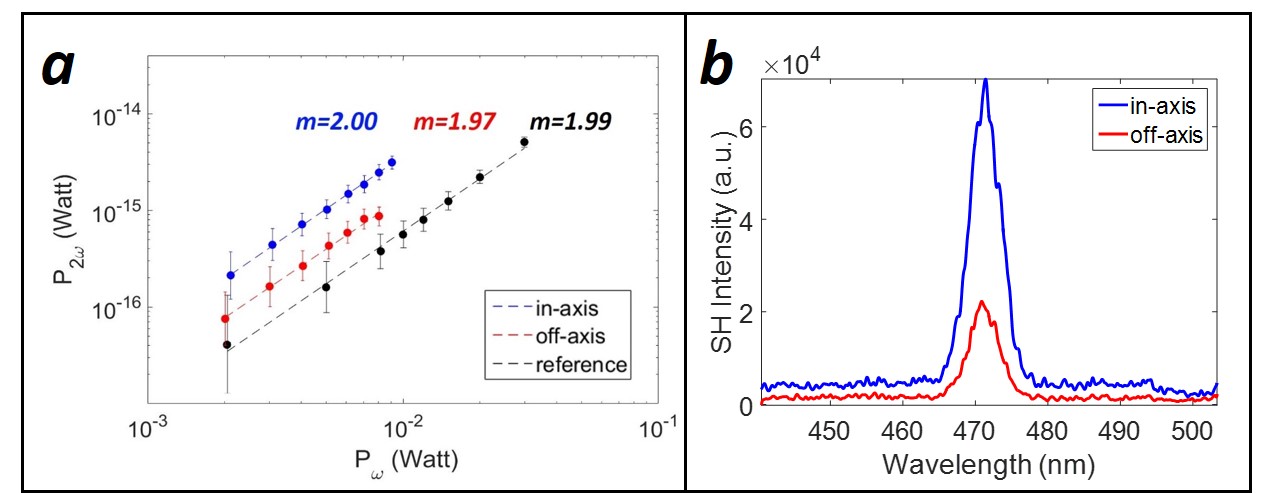


Figure S4: (a) Log-log plot of SHG power vs excitation power for the bare silver film (black) and the studied sample for two orthogonal polarizations (red: off-axis; blue: in-axis polarization). The value of the slope$m$is about 2 as expected and is noted on the graph for each curve with its corresponding color. The *R^2^* values of all fitting curves are greater than 0.99. (b) The SHG spectra are taken from the studied structure for two orthogonal polarizations of the exciting beam (with a fundamental wavelength of 940 nm). Clearly, no additional multi-photon processes are observed, meaning that the observed signals are only due to SHG.

**5. Comparison with a bowtie configuration:**

We studied another structure made of the same basic units, yet in a bowtie configuration. SHG mapping for two orthogonal polarizations, together with a SEM image, are shown in Figure S5. Clearly, the behavior of this structure is very different compared to our studied one. Negligible SHG is emitted from the area in between the two sub-units, unlike the previously studied structure, for which enhanced SHG is emitted from the area in between the two sub-units (Figure 2).


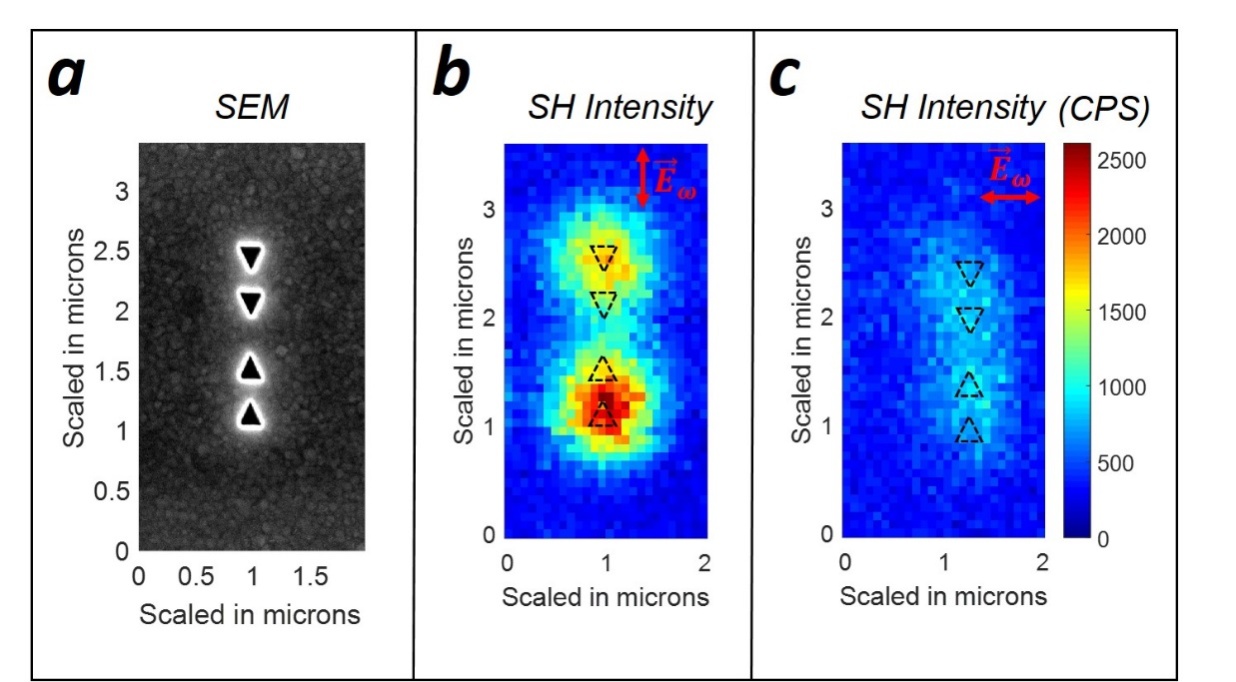


Figure S5: (a) SEM image of the two sub-units in a bowtie-like configuration. The triangular side length is 200 nm, and the tip-to-tip distance is 400 nm. SHG emission mapping for *in-axis* (b) and *off-axis (c)* polarization of the fundamental field*.* For clarity, sketches of the triangles (dashed) are added to guide the eye. Clearly, the emission originates from the two sub-units rather than from the flat area in between.

**6. Intensity and second harmonic efficiency of the studied plasmonic unit:**

For each pulse, the intensity of the irradiated beam $I_{\omega}=\left\langle P_{\omega} \right\rangle/{\nu\cdot\tau\cdot A}=1.3\cdot{10}^{14}$ W m-2 where $\left\langle P_{\omega} \right\rangle=4 mW$ is the average laser power, $\nu=80 MHz$ is the repetition rate of the laser, $\tau\approx100 fs$ is the laser pulse duration, and $A=3.8\cdot{10}^{-12}$ m+2 is the measured spot area at the focus of the laser.^[[1]](#footnote-1)^ The count rate measured by each APD was ~3500 counts per second (CPS) for the in-axis polarization state of fundamental field for the studied plasmonic unit.

To estimate the efficiency, we have taken into account the transmittance efficiency of all the relevant components in our experimental setup. The transmittance of the objective is ~30%^[[2]](#footnote-2)^, the optical elements (dichroic mirror, four filters, polarizing beam splitter, and lenses) are estimated to transmit 70% of the light, the coupling efficiency to the fiber is ~80%, and the APD efficiency at wavelength λ = 470 nm is 60%. The total transmittance efficiency of the system at 470 nm is *t*_e_~ 10%, which gives a corrected measurement rate of 3.5·10^4^ cps for the in-axis polarization state.

We calculated the SH power according to $P_{2\omega}=2\pi\hbar\cdot{c_{ps}}/\left( \lambda\cdot t_{e}\cdot\nu\right)$, where $c_{ps}$ is the measured counts per second rate. Table 1 summarizes the in-axis, off-axis, and flat silver SH responses.

**Table 1.** Second-Harmonic Power, Intensity, and Efficiency

| Effective nonlinear coefficient (1/W) | Maximum conversion efficiency. | SHG peak power (W) | SHG average power (W) | SHG emitted photons (1/s) |  |
| --- | --- | --- | --- | --- | --- |
| *γ =P_2_*_ω_ */P*_ω_^2^ | *η=*〈*P_2_*_ω_〉 */*〈*P*_ω_〉 | *P_2_*_ω_=〈*P_2_*_ω_〉/ (*ν·τ*) | 〈*P_2_*_ω_〉 = *C_ps∙_E_ph_* | *C_ps_* | Symbol/Equation |
| 8.9·10^-15^ | 3.7·10^-12^ | 1.5·10^-9^ | 1.5·10^-14^ | 3.5·10^4^ | Structure are exited with in-axis polarization |
| 3.6·10^-15^ | 1.5·10^-12^ | 6.2·10^-10^ | 6·10^-15^ | 1.4·10^4^ | Structure are exited with off-axis polarization |
| 1.3·10^-15^ | 5.3·10^-13^ | 2.2·10^-10^ | 2.1·10^-15^ | 5·10^3^ | Reference of Flat silver film |

Where *ν,τ* is the repetition rate and pulse duration of the laser respectively, *E_ph_=ω∙ћ* is the photon energy, *ω* is the photon frequency, and *ћ* is Planck's constant.

**7. Polar plots normalization:**

The polar plots shown in Figure 3 are normalized with respect to the SHG to the response of a flat silver film. A flat silver film typically emits ~12000 CPS when is exited with 25mW at 940nm fundamental wavelength. Using the nonlinear response of SHG, the yield is *η=(P_2ω_)/(P_ω_)^2^*. For normalization we divided the yield or the efficiency of the measured values by that of the silver surface.

**8. Quadropolar structure**


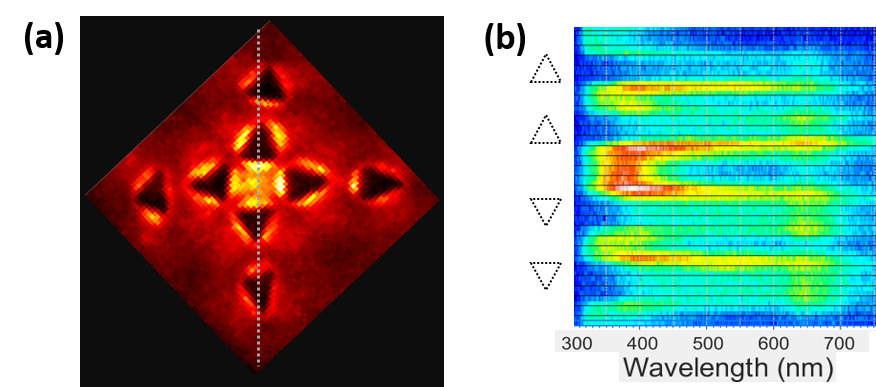


Figure S6. (a) mono CL map of a quadrupolar structure for λ=370 nm ±10 nm. An intense hot spot is observed at the middle. A spectrum is taken along the dash line shown in (b). a clear mode is observed at about 370 nm between the cavities.

**8. The effect of surface quality**

**
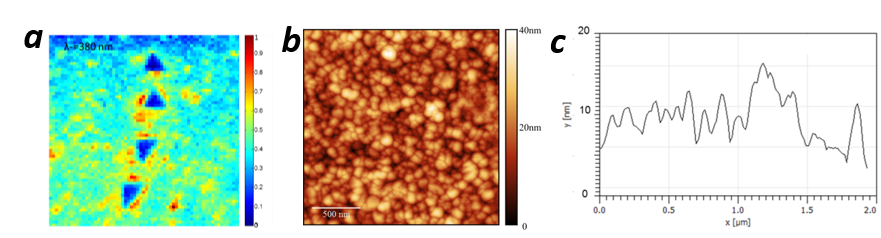
**

Figure S7: CL measurent of the studied structure , but on a relatively rough surface. (a) mono CL image at a wavelength of 380 nm showing that there is no spot at the middle, unlike Figure S6 or Figure 5, for example. (b) and (c) are the AFM scan and the line scan of a rougher silver film comapred to that we studied herein.

1. This value coincides with the calculated spot size for a numerical aperture of 0.5 and a wavelength of 940 nm. [↑](#footnote-ref-1)
2. Calculated with respect to a collection angle of ~1.5 steradian. [↑](#footnote-ref-2)
